# Reductions in Synaptic Vesicle Glycoprotein 2 Isoforms in the Cortex and Hippocampus in a Rat Model of Traumatic Brain Injury

**DOI:** 10.1007/s12035-021-02534-3

**Published:** 2021-08-25

**Authors:** Katherine M. Fronczak, Youming Li, Jeremy Henchir, C. Edward Dixon, Shaun W. Carlson

**Affiliations:** 1 Neurological Surgery, University of Pittsburgh, 4401 Penn Avenue, Pittsburgh, PA 15224, USA; 2 VA Pittsburgh Healthcare System, Pittsburgh, PA, USA

**Keywords:** synapse, traumatic brain injury, neurotransmission, hippocampus, cortex, vesicle

## Abstract

Traumatic brain injury (TBI) can produce lasting cognitive, emotional, and somatic difficulties that can impact quality of life for patients living with an injury. Impaired hippocampal function and synaptic alterations have been implicated in contributing to cognitive difficulties in experimental TBI models. In the synapse, neuronal communication is facilitated by the regulated release of neurotransmitters from docking presynaptic vesicles. The synaptic vesicle glycoprotein 2 (SV2) isoforms SV2A and SV2B play central roles in the maintenance of the readily releasable pool of vesicles and the coupling of calcium to the N-ethylmaleimide-sensitive factor attachment protein receptor (SNARE) complex responsible for vesicle docking. Recently, we reported the findings of TBI-induced reductions in presynaptic vesicle density and SNARE complex formation; however, the effect of TBI on SV2 is unknown. To investigate this, rats were subjected to controlled cortical impact (CCI) or sham control surgery. Abundance of SV2A and SV2B were assessed at 1, 3, 7 and 14 days post-injury by immunoblot. SV2A and SV2B were reduced in the cortex at several time points and in the hippocampus at every time point assessed. Immunohistochemical staining and quantitative intensity measurements completed at 14 days post-injury revealed reduced SV2A immunoreactivity in all hippocampal subregions and reduced SV2B immunoreactivity in the molecular layer after CCI. Reductions in SV2A abundance and immunoreactivity occurred concomitantly with motor dysfunction and spatial learning and memory impairments in the 2 weeks post-injury. These findings provide novel evidence for the effect of TBI on SV2 with implications for impaired neurotransmission neurobehavioral dysfunction after TBI.

## Introduction:

Traumatic brain injury (TBI) can result in cognitive, emotional, and somatic difficulties, including impaired learning, attention, memory, and motor abilities, in clinical settings with features of these impairments recapitulated in experimental models. Patients and clinical reports indicate that persistent cognitive dysfunction can greatly contribute to reduced quality of life of individuals living with a TBI, across the spectrum of injury severity [1-6]. Preclinical research highlights that multiple secondary injury mechanisms can contribute to cellular damage and neurobehavioral impairments, with alterations in neurotransmission and synaptic function having direct implications on neuronal communication and development of cognitive dysfunction. Disruptions in neurotransmission and altered synaptic function have been described using numerous experimental techniques in multiple brain regions, including the hippocampus, cortex, thalamus, and striatum, with changes reported in multiple experimental TBI models of varying injury severity [7-15]. Considering the important role of the somatosensory cortex and the hippocampus and in motor function and learning and memory, considerable effort has been focused on investigating how changes in these regions, including neuronal damage and disruptions in circuitry [9,11,16,17,7,8], contribute to neurobehavioral dysfunction. Previous work from our group described the effect of TBI on altering intrasynaptic vesicular density and distribution in CA1 presynaptic terminals, and hippocampal alterations in the formation of the highly conserved soluble N-ethylmaleimide-sensitive factor attachment protein receptor (SNARE) complexes important for synaptic vesicle docking and neurotransmitter release [18-20].

Synaptic vesicle glycoprotein 2 (SV2), has been identified as a highly conserved vesicular protein that acts as a modulator of synaptic vesicle properties via maintenance of the readily releasable vesicle pool and coupling of calcium during exocytosis to facilitate neurotransmitter release [21-25]. SV2 exists in three isoforms, SV2A, SV2B, and SV2C [26,21,25,27,28]. SV2A is ubiquitously expressed in the brain and throughout the hippocampus, while SV2B is expressed throughout with brain with a few regional exemptions, and is expressed in most hippocampal subregions with notably lower expression in the dentate gyrus granular layer and pyramidal cell layers of CA3, CA2 and CA1 [21,26,29,30]. SV2A and SV2B have been shown to be functionally redundant, both playing an important role in Ca^2+^-mediated exocytosis [21,22]. while less work has been completed to understand the role of SV2C [27,29].

The function of SV2 as a modulator of vesicle fusion has become clear through studies utilizing SV2A and SV2B genetically deficient mice. Taken together, knockout mouse models have identified two critical functions for SV2 during vesicle exocytosis, with the first being that SV2 is required for the maintenance of the readily releasable pool and vesicle distribution in the active zone [25,24,31], and the second being that SV2 is upstream of SNARE complex formation to couple calcium signaling to conformational changes in the complex, facilitating vesicular fusion and neurotransmitter release [25,31,22,27,23]. Based upon the roles of SV2A and SV2B isoforms in synaptic function, both isoforms were examined in the cortex and hippocampus. Considering that reductions in the presynaptic vesicular pool and impaired SNARE complex formation have both been reported in the weeks following TBI [18,19,32,20], it is plausible a post-traumatic change in the abundance of SV2 isoforms could contribute to synaptic dysfunction.

The goal of the current study was to evaluate the effect of traumatic brain injury, using a rat model of controlled cortical impact injury (CCI), on SV2A and SV2B abundance in the cortex and hippocampus. We hypothesized CCI would reduce the abundance of cortical and hippocampal SV2A and SV2B isoforms in the days to weeks post-injury. To test this, rats were subjected to CCI injury, and protein abundance was assessed at 1, 3, 7, or 14 days post-injury. Immunoblotting was completed to evaluate SV2A and SV2B protein abundance in the cortex and hippocampus. Immunohistochemistry and subregional quantitative intensity measurements were completed to identify hippocampal changes in SV2 isoform immunoreactivity. These findings provide novel evidence for reduced cortical and hippocampal SV2 isoform abundances in the days and weeks following TBI.

## Methods and Materials

### Animals

All experimental procedures were approved by the University of Pittsburgh Institutional Animal Care and Use Committee in accordance with the guidelines established by the National Institutes of Health in the Guide for the Care and Use of Laboratory Animals. Adult male Sprague Dawley rats (Envigo, Indianapolis, IN), aged 10–15 weeks and weighing 275–375 g, were housed up to 2 rats per cage in the University of Pittsburgh vivarium with a 12:12 light/dark photoperiod (lights on at 7:00 am) and with *ad libitum* access to food and water. A total of 82 rats were utilized in the current study, and the breakdown of the groups is detailed below for the multiple outcomes assessed.

### Controlled cortical impact

In a ventilated anesthesia chamber, rats were anesthetized using 4% isoflurane with a 2:1 N_2_O:O_2_ mixture. After endotracheal intubation, the rats were placed on a mechanical ventilator (683 Small Animal Ventilator, Harvard Apparatus) and anesthesia was maintained with a 2% isoflurane mixture. Rats were placed in a stereotaxic frame and body temperature was monitored by a rectal thermistor probe, with body temperature maintained at 37°C using a computer-controlled heating pad. Following a midline incision on the surgically prepared scalp, the soft tissues were reflected and a 7 mm craniotomy was completed, using a dental drill, between bregma and lambda and centered 5 mm lateral of the sagittal suture to expose the dura mater of the right parietal cortex. Rats were randomly assigned to receive controlled cortical impact (CCI) injury or sham control surgery, as previously described [32,33]. Sham animals received all surgical procedures except the induction of the CCI injury. CCI injury was completed using a small bore (1.975 cm) double-acting stroked-constrained pneumatic cylinder with a 5.0 cm stroke. A beveled metal impactor tip, 6 mm in diameter, was set to produce a 2.7 mm tissue deformation at a velocity of 4 m/s with a dwell time of 100 msec. At 5 minutes following CCI injury or sham surgery, animals received an intraperitoneal injection of 1ml/kg USP saline, which continued daily until the time of euthanasia. Following the CCI injury or sham surgery, the scalp was sutured, anesthesia stopped, and the righting time of each animal was monitored. After the rats became ambulatory, they were returned to their home cage. Animals are monitored daily for changes in post-operative health evaluating multiple parameters of health and wellbeing.

### Vestibular motor function

A total of 22 rats (n=11/group) were utilized to evaluate vestibular motor function and spatial learning and memory similar to previously described [19,32]. A modified beam walking task was utilized to evaluate fine motor components of vestibular motor function. Prior to injury, animals were trained to escape a loud pink noise by traversing a narrow wooden bean (2.5 cm x 100 cm) to enter a darkened goal box at the end of the beam. Four pegs (4cm high and 3mm in diameter) were equally spaced across the length of the beam to increase the difficulty of the test. If the rats fell off the beam or did not traverse the beam in the allotted 60 second period, the noise was stopped, and the animal was placed in the goal box. Performance was assessed by recording the average latency to traverse the beam, as well as distance traveled on days 1-5 post-injury, with 3 trails per day. The distance traveled is also expressed as a score from 0 to 5, with 0 indicating an inability to move beyond the starting location, 1 to 4 corresponding to segments of 20, 40, 60, or 80 cm from the start point respectively, and 5 indicating that the animal traversed the entire length of the beam (100 cm) and entered the goal box.

### Spatial learning and memory

Spatial learning and memory were evaluated in the same rats subjected to vestibular motor testing using the Morris water maze (MWM) task using a video-tracking system (AnyMaze, Stoelting, Inc., Wood Dale, IL, USA) as previously described [19,32]. A 180 cm circular tank (45 cm high) was filled with 26±1°C water to a height of 30 cm to conceal a transparent circular platform (10 cm in diameter and 29 cm high) in a fixed location positioned 45 cm from the wall. Visual cues located on the walls outside the tank aid in locating the escape platform. Testing began on day 9 post-injury, without previous exposure or training to the MWM task, and continued for 5 days (days 9-13 post-injury) with each animal completing four trials per day. Rats were randomly placed in the water against the wall and released to swim in the tank to find the hidden platform in a 120 second period. In the event the animal was unable to locate the platform within the allowed time, it was manually directed to the platform. The rat remained on the platform for 30 seconds and were placed in an incubator between trials. Following a 4 minute intertrial interval, the subsequent trial was initiated. On day 14 post-injury, the animal was tested using a probe trial paradigm in which the hidden platform was removed. The location of the hidden platform was designated as platform, with additional target zones of concentric inner and outer rings, and the remaining area of the quadrant. Probe trial times were utilized to assess probe trial performance between the group. A breakdown of the percent time spent in each region was also generated to assess the composition of time spent in the target zones during the allotted probe trial period.

### Tissue preparation for western blot

A total of 48 rats (n=6 per group at each time point) received an overdose of Fatal-plus (100mg/kg sodium pentobarbital) and the brains rapidly dissected at 1, 3, 7 or 14 days post-injury. After removal of the brain, the ipsilateral hippocampus and cortex were rapidly dissected on a chilled ice plate, immediately snap frozen with liquid nitrogen and tissues stored at −80°C. Samples were homogenized using a lysis buffer (0.1 M NaCl, 0.01 M Tris-Cl (pH 7.6), 0.001 M EDTA) with protease inhibitor cocktail (Pierce, Rockford, IL, Cat. No. 1861281). The homogenized whole cell lysates were centrifuged at 12,000xg at 4°C for 30 minutes and the supernatants collected. The protein concentration for each lysate was determined using a BCA assay (Thermo Scientific, Pittsburgh, PA) and a 96 well microplate reader (Biotek, Winooski, VT).

### Western blot

To evaluate SV2A and SV2B abundance in ipsilateral hippocampal and cortical tissues from CCI-injured or sham rats, 30 μg of protein for each prepared lysate were subjected to sodium dodecyl sulfate polyacrylamide gel electrophoresis (SDS-PAGE) through a 10% gel to separate protein samples and molecular weight markers (Bio-Rad, Hercules, CA). Resolved proteins were electrophoretically transferred to a PVDF membrane. The membrane was blocked in 5% non-fat dry milk in 0.1 M PBS with 0.1% Tween-20 (PBST) at room temperature for one hour, and immunolabeled using a commercially available antibody specific to SV2A (rabbit polyclonal 1:2,000, Abcam, Cat. No. 32942) or SV2B (rabbit polyclonal, 1:2000, Synaptic systems (SySy), Cat. No. 119103) at 4°C overnight followed by respective anti-rabbit or anti-mouse immunoglobulin G conjugated to peroxidase (1:10,000; Pierce, Rockford, IL) at room temperature for one hour. Proteins were visualized with a chemiluminescence detection system (SuperSignal, Pierce, IL). To normalize for protein loading, the membranes were stripped and re-blotted with mouse anti-β-actin monoclonal antibody (1:20,000, Sigma, St. Louis, MO, Cat. No. A5316). The CCI-injured and sham control tissue were loaded together in the same gel for comparison. Blots were developed using electrochemicalilluminscence (Pierce), imaged (Chemidoc, Biorad) and band intensity quantified using ImageJ software (NIH). Optical density of each isoform, normalized to its respective actin loading, are reported as the percentage of mean sham control at every time point. Data are expressed as the group means ± standard error of the mean (SEM).

### Tissue preparation for immunohistochemistry

A total of n=12 rats (n=6 per group) were sacrificed at 14 days following CCI or sham control surgery, a time point of reduced SV2 isoform abundance currently shown by immunoblot, and a time point of impaired SNARE complex formation following CCI [20]. Animals received an overdose of Fatal-plus (intraperitoneally, 100 mg/kg, sodium pentobarbital (Fatal-Plus), Vortech Pharmaceuticals, Dearborn, MI) and then were transcardially perfused with saline, followed by 10% neutral buffered formalin (Fischer Scientific, Waltham, MA). The brains were post-fixed for an additional 24 hours in 10% neutral buffered formalin and then cryoprotected with 30% sucrose in 0.1 M phosphate buffered saline (PBS) for 48 hours at 4°C. The brains were frozen in Tissue-Tek OCT compound (Sakura Finetek, Torrance, CA) and cut into 35 μm thick coronal sections using a cryostat (Leica Microsystems Inc., Buffalo Grove, IL). Hippocampal tissue sections, ranging from −3.2 mm to −4.0 mm bregma [34], encompassing the site of injury, were selected for immunohistochemical staining and utilized for intensity measurements.

### Immunohistochemistry

Immunohistochemical staining for SV2A and SV2B were completed at 14 days post-injury. Immunohistochemical staining was completed in 24 well plates on free-floating tissue sections utilizing one section from each animal, for each isoform, focused at the CCI injury site incorporating bregma levels −3.2 to −4.0 mm [34]. The sections were rinsed with 0.1M tris-buffered saline (TBS) buffer and blocked with 10% normal goat serum and 0.1% Triton X-100 in 0.1M TBS (TBS-T) for 1 hour. Sections were incubated overnight at 4°C with anti-SV2A antibody (rabbit polyclonal 1:1,000, SySy, Cat. No. 119003) or anti-SV2B antibody (rabbit polyclonal 1:1,000, SySy, Cat. No. 119103). The following day, sections were rinsed with TBS-T, incubated with horseradish peroxidase secondary antibody and rinsed with TBS-T to prepare for substrate development. For each isoform, all sham control and CCI-injured tissues were run simultaneously, diaminobenzidine (DAB) was added, and the reaction development time was tightly controlled to ensure equal diaminobenzidine (DAB) substrate exposure time between all sections within each isoform set for measurements of pixel intensity (SK-4100, Vector Laboratories, Burlingame, CA). Sections were mounted on Superfrost Plus slides (Fischer Scientific), and were cover-slipped using Permount medium (Fischer Scientific).

### Quantification of SV2 Isoform Immunoreactivity

To quantify pixel intensity differences in immunohistochemical staining of sham and CCI-injured brains, images of stained hippocampal slices were acquired at 10x magnification and merged into one image using a C2 Nikon 90i microscope. Images for SV2A and SV2B (n=6 sham and n=6 injured per isoform) were converted to 32-bit grayscale and inverted to prepare for mean pixel intensity measurements using ImageJ. Hippocampal subregions of the molecular layer, hilus, granular layer, CA1, CA2 and CA3 for each section were identified and quantification guided as defined by the rat brain atlas [34]. The polygon tool was used to delineate the described subregions of the ipsilateral and contralateral hippocampus separately to complete measurements of mean pixel intensity for each defined subregion. The mean pixel intensity for the medial corpus callosum was subtracted from the mean pixel intensity of each subregion in the same section to normalize background staining intensity for every section. Mean pixel intensity measures of each subregion on the ipsilateral and contralateral hemispheres were averaged separately. For each subregion, an average group mean of normalized pixel intensities were calculated and normalized as a percentage of sham intensity for ipsilateral and contralateral separately.

### Statistical Analysis

All quantification was completed by an investigator blinded to the injury status of each animal. Data are presented as mean ± standard error of the mean (SEM). Immunoblot data for each isoform and region were compared by two-way analysis of variance (ANOVA) comparing time and injury status. When appropriate, a Sidak post hoc multiple comparisons test was completed. Mean pixel intensity measurements of immunohistochemical staining, were analyzed by Student’s t-tests assessing the difference between sham and CCI-injured groups for each subregion of each hemisphere. Statistical comparisons of beam walking latency, beam walking score and spatial acquisition latency, swim speed and escape distance were completed using a two-way ANOVA followed by Sidak post-hoc multiple comparisons test when appropriate. Probe trial time and breakdown of target zones were statistically compared using a Student’s t-test. Statistical tests were completed using Graphpad (Graphpad version 9, La Jolla, CA). A *p* value < 0.05 was considered statistically significant for all tests.

## Results:

**Figure 1:**
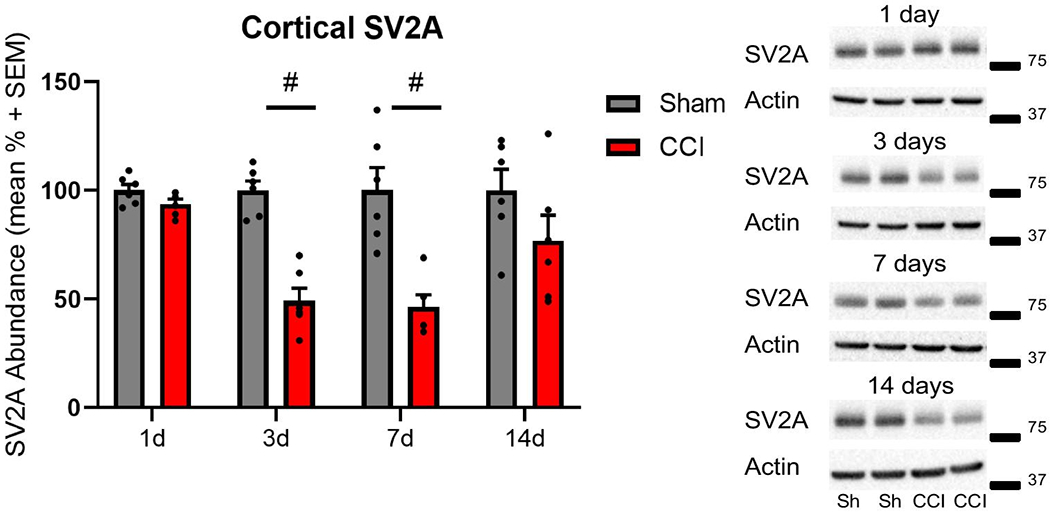
Controlled cortical impact (CCI) reduced cortical SV2A abundance post-injury. Representative western blot images of SV2A (83 kDa, marker is 75 kDa) and actin (42 kDa, marker is 37 kDa) in cortical whole cell lysates following CCI or sham (Sh) control surgery. Semiquantitative measurements of cortical homogenates after CCI revealed significantly reduced SV2A abundance at 3 and 7 days post-injury (# p<0.01). SV2A was normalized to actin (n=6 per group per time point).

**Figure 2:**
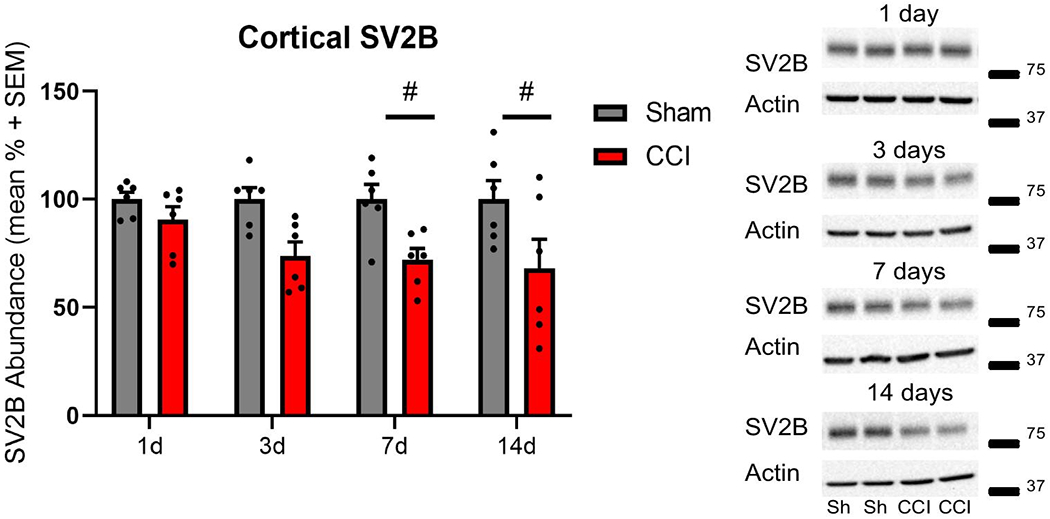
Controlled cortical impact (CCI) reduced cortical SV2B abundance post-injury. Representative western blot images of SV2B (88 kDa, marker is 75 kDa) and actin (42 kDa, marker is 37 kDa) in cortical whole cell lysates following CCI or sham (Sh) control surgery. Semiquantitative measurements of cortical homogenates after CCI revealed significantly reduced SV2B abundance at 7 and 14 days post-injury (# p<0.05). SV2B was normalized to actin (n=6 per group per time point).

In the cortex, SV2A protein abundance was significantly reduced at 3 and 7 days following CCI, compared to sham control at each time point (two-way ANOVA, main injury effect p<0.001 F(1, 40) =41.44; main time effect p<0.01 F(3, 40) = 4.784; interaction p<0.01 F(3, 40) = 4.769; post hoc t-test p<0.01; Fig. 1). There was a trend toward reduced SV2A abundance at 14 days post-injury, but did not reach significance. SV2B was significantly reduced at 7 and 14 days after CCI, as compared to sham control at each time point (two-way ANOVA, main injury effect p<0.001 F(1, 40) = 20.83; main time effect p=0.46 F(3, 40) = 0.883; interaction p=0.046 F(3, 40) = 0.888; post hoc t-test p<0.05; Fig. 2). There was a trend toward reduced SV2B abundance at 3 days post-injury, but did not reach significance.

**Figure 3:**
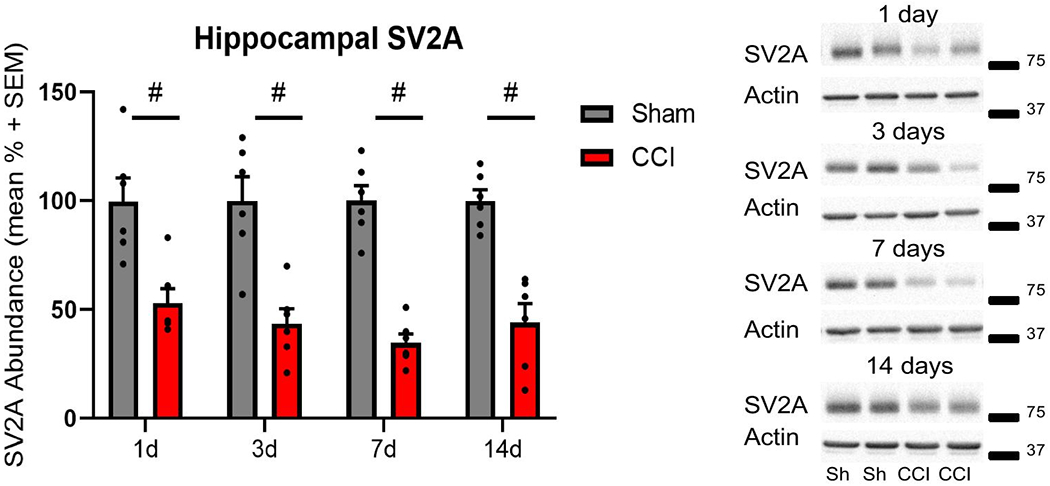
Controlled cortical impact (CCI) reduced hippocampal SV2A abundance post-injury. Representative western blot images of SV2A (83 kDa, marker is 75 kDa) and actin (42 kDa, marker is 37 kDa) in hippocampal whole cell lysates following CCI or sham (Sh) control surgery. Semiquantitative measurements of hippocampal homogenates revealed significantly reduced SV2A abundance at all time points assessed (# p<0.01). SV2A was normalized to actin (n=6 per group per time point).

**Figure 4:**
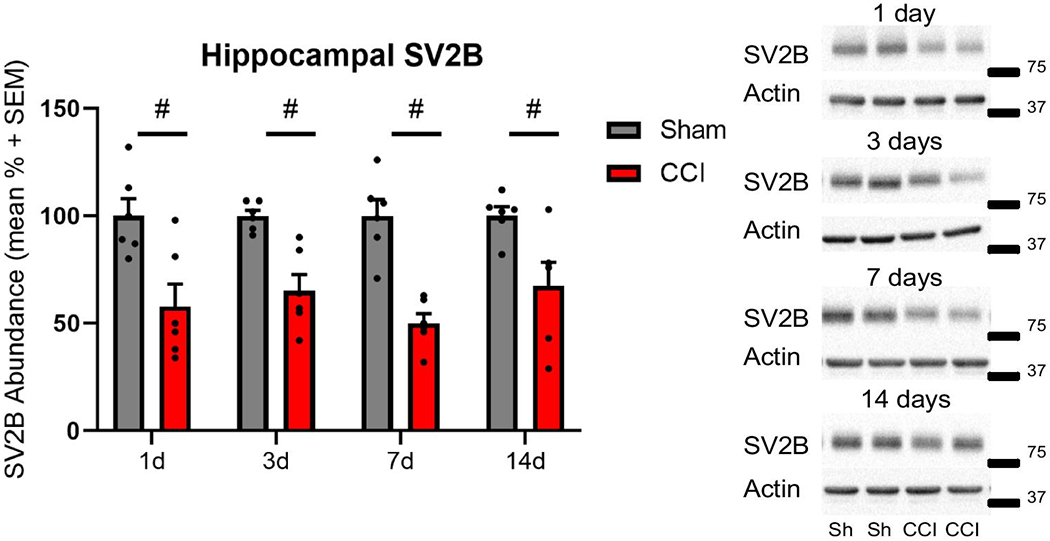
Controlled cortical impact (CCI) reduced hippocampal SV2B abundance post-injury. Representative western blot images of SV2B (88 kDa, marker is 75 kDa) and actin (42 kDa, marker is 37 kDa) in hippocampal whole cell lysates following CCI. Semiquantitative measurements of hippocampal homogenates revealed significantly reduced SV2B abundance at all time points assessed (# p<0.05). SV2B was normalized to actin (n=6 per group per time point).

In the hippocampus, SV2A abundance was significantly reduced at 1, 3, 7 and 14 days following CCI, as compared to sham control at each time point (two-way ANOVA, main injury effect p<0.001 F(1, 40) = 103.1; main time effect p=0.73 F(3, 40) = 0.434; interaction p=0.71 F(3, 40) = 0.468; post hoc t-test p<0.001; Fig. 3). Similar to SV2A isoform, SV2B showed significant reductions at all time points assessed after CCI, as compared to sham control (two-way ANOVA, main injury effect p<0.001 F(1, 40) = 56.46; main time effect p=0.64 F(3, 40) = 0.564; interaction p=0.65 F(3, 40) = 0.554, post hoc t-test p<0.001; Fig. 4).

**Figure 5:**
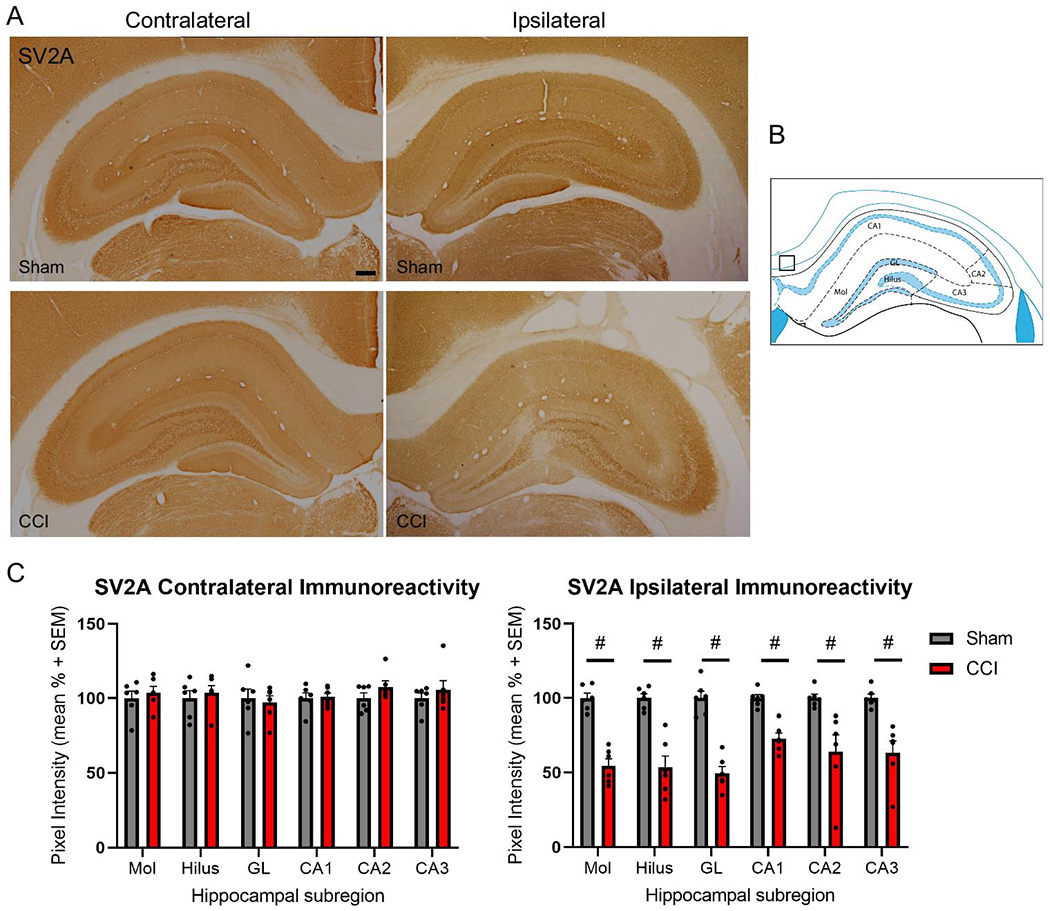
Controlled cortical impact (CCI) reduced ipsilateral subregional hippocampal SV2A immunoreactivity at 14 days post-injury. Representative images of SV2A immunoreactivity in the contralateral and ipsilateral hippocampus at 14 days following sham control surgery or CCI (A). Mean pixel intensity measures revealed no differences in SV2A immunoreactivity in the contralateral hippocampal subregions of sham control and CCI-injured rats (C). Mean pixel intensity measures revealed significant reductions in all ipsilateral hippocampal subregions assessed (#p<0.01; C). Subregional analyses were completed in the molecular layer (Mol), hilus, dentate gyrus granular layer (GL), CA1, CA2 and CA3 and normalized to the mean pixel intensity within the box (B), according to the rat brain atlas [34]. Scale bar is equal to 250 μm (n=6 per group).

**Figure 6:**
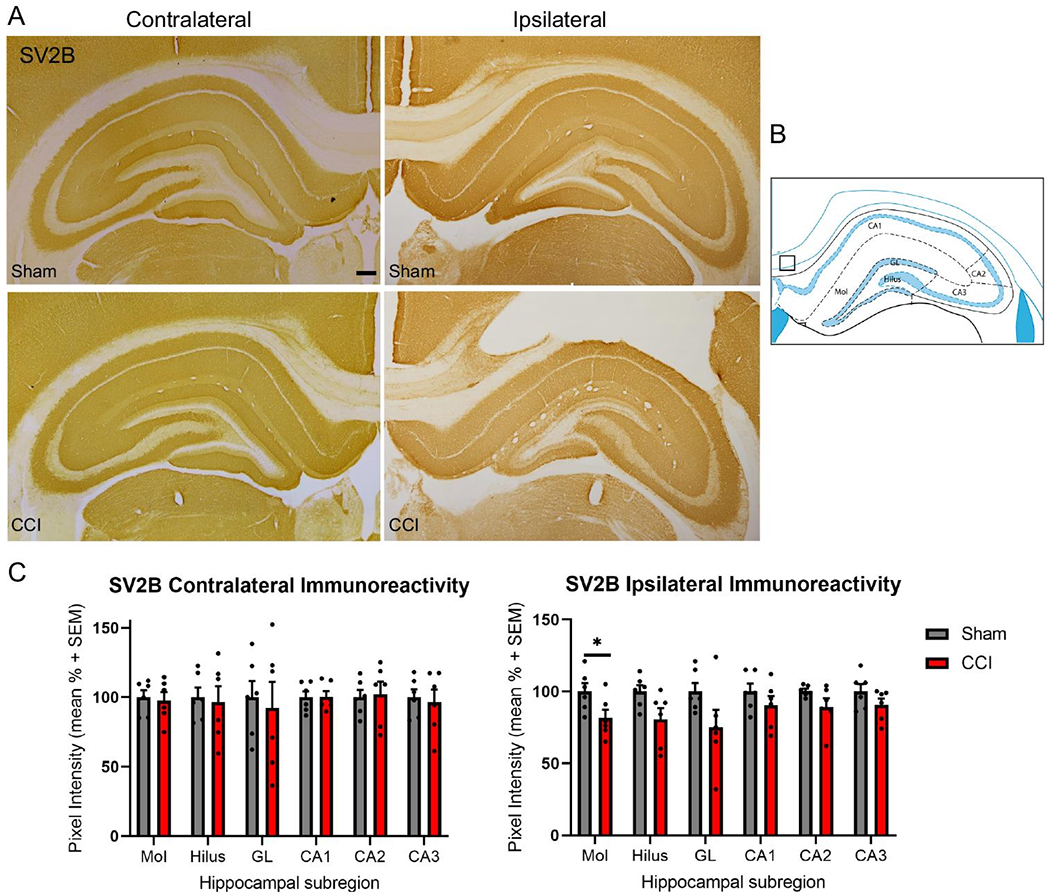
Controlled cortical impact (CCI) reduced ipsilateral hippocampal SV2B immunoreactivity in the molecular layer at 14 days post-injury. Representative images of SV2B immunoreactivity in the contralateral and ipsilateral hippocampus at 14 days following sham control surgery or CCI (A). Mean pixel intensity measures revealed no differences in SV2B immunoreactivity in the contralateral hippocampal subregions of sham control and CCI-injured rats (C). Mean pixel intensity measures revealed significant reductions in the molecular layer (#p<0.01; B). Subregional analyses were completed in the molecular layer (Mol), hilus, dentate gyrus granular layer (GL), CA1, CA2 and CA3 and normalized to the mean pixel intensity within the box (B), according to the rat brain atlas [34]. Scale bar is equal to 250 μm (n=6 per group).

To investigate potential differences in hippocampal SV2 isoform abundances after TBI, both as a result of injury and differential expression patterns, immunoreactivity for both SV2A and SV2B were assessed in hippocampal subregions. The 14 day time point was selected as impaired hippocampal evoked neurotransmitter release, reduced SNARE complex formation and neurobehavioral dysfunction have been reported at this time point following CCI [32,20,35]. The dentate gyrus granular layer, hilus, molecular layer, CA1, CA2 and CA3 were defined using the rat brain atlas [34] (Fig 5B, 6B), and analyzed for mean pixel intensity to examine subregional changes in SV2 isoform abundance. Measurements of SV2A immunoreactivity revealed significant reductions in all ipsilateral hippocampal subregions at 14 days post-injury (t-test at each subregion, p<0.05; Fig. 5A, C). Assessment of contralateral subregional SV2A immunoreactivity revealed no significant differences between sham control and CCI-injured rats for all subregions (Fig. 5A, C). Measurements of ipsilateral SV2B immunoreactivity revealed significant reductions in immunoreactivity in the molecular layer after CCI (t-test, p<0.05, Fig. 6A, C), with modest trends toward reductions in the hilus and granular layers, but these differences did not reach significance (p=0.054 and p=0.09, respectively). Assessment of contralateral subregional SV2B immunoreactivity revealed no significant differences between sham control and CCI-injured rats for all subregions (Fig. 6A, 6C).

**Figure 7:**
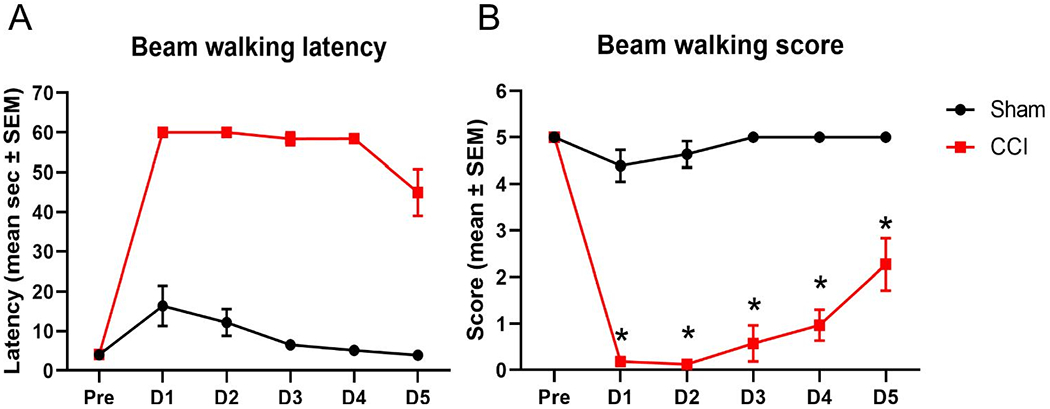
Controlled cortical impact (CCI) impaired beam walking performance in the 5 days post-injury. Assessment of motor function in the beam task revealed significant impairment in beam walking latency in CCI-injured rats (n=11) compared to sham control rats (n=11) over the 5 day testing period (two-way ANOVA, main injury effect p<0.0001; A). Scoring of the beam walking task demonstrates reduced score reflective of impaired motor function in CCI-injured rats, compared to sham control rats on days 1, 2, 3, 4 and 5 post-injury (*p<0.01; B).

Acute neurobehavioral testing of vestibular motor function was completed in the first 5 days post-injury to determine if SV2 isoform abundance changes occur at time points associated with impaired motor function after CCI. In the beam walking task, CCI-injured animals exhibited a significant increase in the latency over the five day period, compared to sham control animals (two-way ANOVA; main injury effect p<0.0001 F(1, 20) = 466.0; time main effect p<0.001 F(2.204, 44.08) = 8.195; interaction p=0.1 F(4, 80) = 2.009; Fig. 7A). Assessment of beam walking scores revealed a significant reduction in performance of CCI-injured animals compared to sham control animals on all 5 days post-injury (two-way ANOVA; main injury effect p<0.0001 F(1, 20) = 322.0; time main effect p<0.0001 F(2.852, 57.04) = 8.655; interaction p<0.01 F(4, 80) = 3.895; post-hoc t-test p<0.01; Fig. 7B).

**Figure 8:**
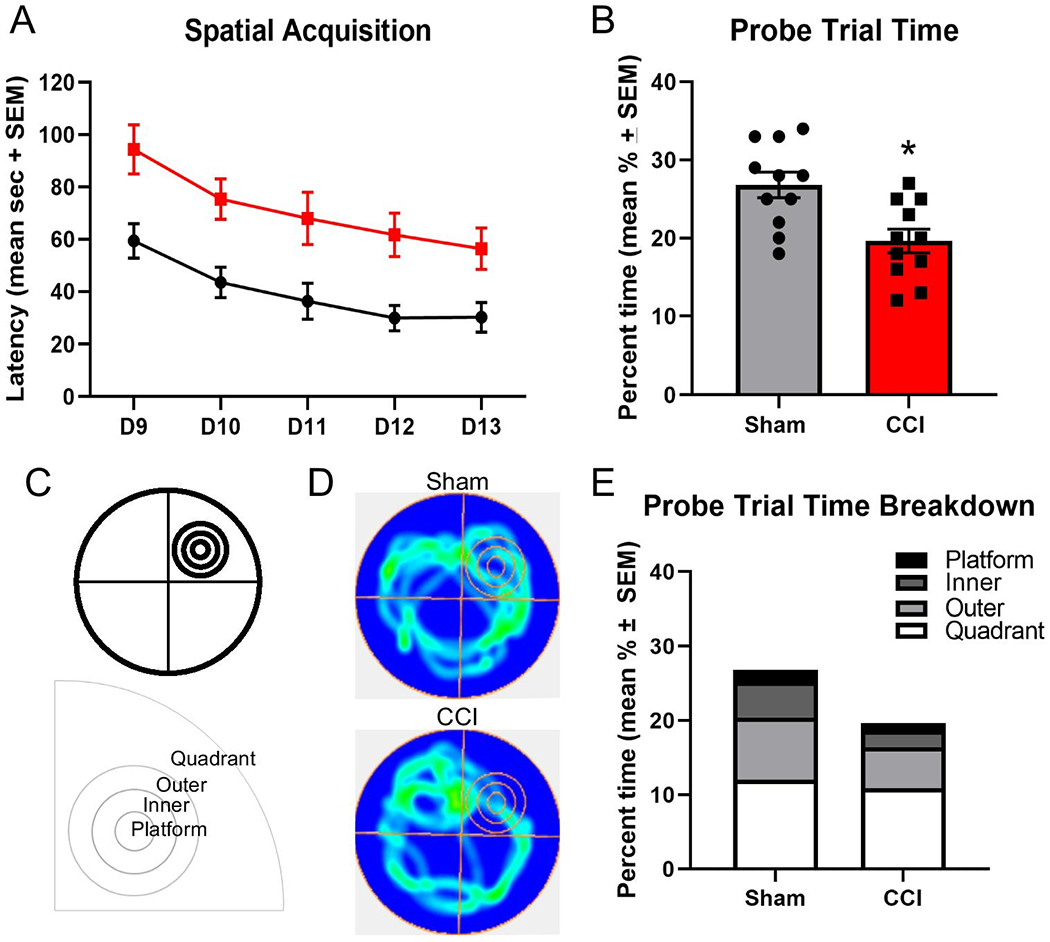
Controlled cortical impact (CCI) impaired Morris water maze spatial learning and memory performance on days 9-14 post-injury. Assessment of spatial learning on days 9-13 post-injury revealed a significant increase in learning latency in CCI-injured rats (n=11) compared to sham control rats (n=11) over the 5 day testing period (main injury effect, p<0.001; A). Testing of spatial memory using the hidden platform probe trial on day 14 post-injury, revealed CCI-injured rats spent significantly less percentage of the 60 second testing period in the target quadrant, as compared to sham control rats (*p<0.005; B). Additionally, a further compositional breakdown was completed to gain resolution of time spent in the platform, inner ring, outer ring, and remainder of the target quadrant (C). Heat map (D) and breakdown of the subdivided quadrant (E) highlights that while sham control and CCI-injured rats spent significantly comparable percentage of time in the quadrant, CCI-injured rats spent significantly less time in the outer ring, inner ring and platform as compared to sham control rats (E). Data are presented as mean ± SEM.

Spatial learning and memory were assessed using the MWM task starting on day 9 post-injury to assess if SV2 isoform abundance changes post-injury occurred concomitantly with spatial learning and memory impairments after CCI. CCI-injured animals exhibited a significant increase in spatial acquisition learning latencies over the testing period from 9 to 13 days post-injury, as compared to sham control animals (two-way ANOVA; main injury effect p<0.0005 F(1, 20) = 18.25; main time effect p<0.0001 F(3.360, 67.20) = 9.903; interaction p=0.968 F(4, 80) = 0.1386; Fig. 8A). Assessments of swim speed during spatial acquisition testing revealed CCI-injured rats exhibited significantly increased swim speeds over the testing period (two-way ANOVA; main injury effect p<0.05 F(1,21) = 4.436; D9, 21.1 ± 0.8 cm / sec; D10, 20.1 ± 0.9 cm / sec; D11, 21.1 ± 0.9 cm / sec; D12, 21.5 ± 1.0 cm / sec; D13, 21.9 ± 1.1 cm / sec), as compared to sham control rats (D9, 20.1 ± 0.8 cm / sec; D10, 20.2 ± 1.2 cm / sec; D11, 20.0 ± 1.1 cm / sec; D12, 18.8 ± 1.0 cm / sec; D13, 17.9 ± 1.0 cm / sec). Assessment of escape distance revealed a significant increase in distance traveled for CCI-injured rats over the testing period (two-way ANOVA; main injury effect p<0.001 F(1,21) = 25.73; main time effect, p<0.01 F(4,84) = 11.70; D9, 21.4 ± 2.2 m; D10, 16.9 ± 1.6 m; D11, 14.1 ± 1.8 m; D12, 13.3 ± 1.8 m; D13, 12.4 ± 1.3 m), as compared to sham control rats (D9, 12.7 ± 1.3 m; D10, 9.9 ± 1.4 m; D11, 7.9 ± 1.4 m; D12, 6.7 ± 1.1 m; D13, 6.3 ±1.0 m). In the hidden platform probe trial on day 14 post-injury, CCI-injured animals spent significantly less time in the total target area comprising the SW quadrant, outer ring, inner ring, and platforms combined, as compared to sham control animals (t-test, p<0.005; 26.8±1.6, 19.6±1.5 seconds, respectively; Fig. 8B and 8C), and the observed swim patterns during the task (Fig. 8E). A breakdown of the percentage of time spent in the target zones reveals CCI-injured animals and sham control animals spent statistically similar percentage of time in the quadrant (t-test, p=0.39; 12.0±0.7% and 10.9±1.0%, respectively); however, CCI-injured animals spent significantly less percentage of the time, as compared to sham control animals, in the outer ring (p<0.005; 8.4±0.6% and 5.6±0.8%, respectively), inner ring (t-test, p<0.0005; 4.8±0.4% and 2.2±0.4%, respectively) and platform (t-test, p<0.05; 1.6±0.2% and 0.9±0.2%, respectively; Fig 8E). The latency to first entry to the platform zone was modestly increased in CCI-injured animals (33.3±4.9 seconds) as compared to sham control animals (25.3±4.3 seconds), but this did not reach significance (t-test, p=0.23).

## Discussion:

The goal of the current study was to examine the effect of TBI on the abundance of SV2A and SV2B isoforms over a time course of 1 day to 14 days post-injury. We demonstrate the effect of TBI on SV2A and SV2B cortical and hippocampal protein abundances during the two weeks following TBI, concomitant with impairments in motor and cognitive performance in beam walking and MWM tasks. Immunohistochemical intensity measurements revealed significant changes in SV2A and SV2B immunoreactivity in hippocampal subregions at 14 days post-injury. The current findings provide new insight into alterations in SV2 isoforms, proteins identified to be important for neurotransmission and synaptic function, in the weeks following TBI. In light of the established roles of SV2A and SV2B in intrasynaptic vesicle dynamics and exocytosis [24,36,25], post-traumatic changes in SV2A and SV2B could contribute to synaptic dysfunction and may underlie TBI-induced alterations in vesicular properties and impaired SNARE complex formation previously described in the weeks following CCI [18,32,20].

SV2A knockout and SV2A/B double knockout mouse models have revealed two key modulatory synaptic functions, including regulation of the size and organization of the vesicular readily releasable pool (RRP), and the coupling of calcium signaling to conformational changes in the SNARE protein [25,22,24,31]. While there appears to be some degree of functional redundancy between SV2A and SV2B [27,23], these knockout studies have suggested that changes in SV2A leads to greater functional consequences. SV2A knockout and SV2A/B double knockout mice exhibit stunted growth, severe seizures, and die in the weeks postnatally without exhibiting abnormalities in overt brain morphology, while SV2B knockout mice are phenotypically normal without the presence of seizure activity [27,36,22]. This phenotype in SV2A knockout mice is not due to changes in number or structure of synapses [36], but is characterized by reductions in the RRP of vesicles and in the formation of SNARE complexes in the synapse [25]. Additionally, targeted deletion of SV2B results in altered regulation of resting and evoked presynaptic calcium signaling in mouse rod bipolar neurons [23]. Taken together, these studies support the role of SV2 isoforms in synaptic vesicular pool maintenance and exocytosis for the release of neurotransmitters.

In the context of TBI, the effect of injury on SV2 isoform abundance is largely unknown. A handful of microarray studies have shown reductions in SV2A mRNA in the hippocampus at 1 year following fluid percussion injury, and reductions in SV2B mRNA in both the hippocampus at 24 hours following blast injury and in the site of cortical contusion at 72 hours following CCI [37-39]. The current immunoblot and immunohistochemical findings suggest that the SV2A isoform may exhibit a greater extent of reduction in multiple hippocampal subregions than the SV2B isoform, but this study was not designed to delineate this question of the causative role of SV2A or SV2B reductions after TBI. While it is speculative, the SV2A and SV2B knockout studies suggest that SV2A may play a more prominent role in maintenance of the synaptic vesicular pool and SNARE complex formation than SV2B [22,24,25,27,30,31]. However, to answer this question, targeted investigation is needed to understand how post-traumatic alterations in either SV2A or SV2B can contribute to altered synaptic vesicular pool maintenance, SNARE complex formation, and potentially the development of post-traumatic epileptogenic changes as described in the weeks to months following CCI [10,16,40-42].

Under normal conditions, SV2A is expressed ubiquitously throughout the hippocampal synaptic layers and in the cortex [21,29]. In the current study, we provide novel evidence of injury-induced hippocampal alterations in SV2 isoforms, with reduced SV2A and SV2B levels observed at all time points assessed. One of the central functions of SV2A and SV2B is the regulation of the RRP of vesicles, as demonstrated in genetic mouse models and cultured neurons [24,25]. We have previously shown CCI results in a reduction in intrasynaptic vesicle density and altered vesicular distribution of presynaptic terminals in CA1 of the hippocampus at 7 days post-injury [20]. This pathological change in presynaptic vesicular docking has also been observed in cortical synapses at 30 days after low-intensity blast injury [43], suggesting synaptic vesicle alterations occur across a broad spectrum of TBI severities and modalities.

Our SV2A immunohistochemical staining demonstrated widespread hippocampal expression in sham control conditions, and following the CCI, loss of SV2A immunoreactivity was observed throughout all of the assessed hippocampal subregions. Consistent with SV2B expression patterns, reduced granular layer and pyramidal cell expression was observed in sham control conditions, but following CCI, only the molecular layer, and to a less extent the hilus, showed reduced SV2B immunoreactivity. Future studies are needed to better understand potential reasons for immunoreactivity differences between SV2A and SV2B isoforms following injury. Interestingly, SV2A and SV2B exhibit differences in neuronal subtype expression patterns, with the SV2A isoform expressed in glutamatergic and GABAergic neurons while SV2B is expressed only in glutamatergic neurons [21,44]. Additional work is needed to better understand the temporal time course of changes in SV2 subregional immunoreactivity and if the observed changes are reflective of differential responses in neuronal subtypes. If differential SV2 responses occur in varying neuronal subtypes, it is possible the influence of TBI on SV2A could contribute to vulnerability of GABAergic neurons as the numbers of multiple interneuron subtypes are reduced in the dentate gyrus, hilus and other subregions of the hippocampus at 30 days following CCI [45].

In the model of CCI, the temporal association of SV2 isoform reductions in the molecular layer and CA1, as observed in the current study, may directly contribute to the reduced vesicular density and altered distribution of vesicles reported within 150 nm of the active zone [20], inclusive of the readily releasable pool and docked vesicles known to be regulated by SV2 [24,25]. The formation of the SNARE complex, the protein machinery critical for vesicular docking and neurotransmitter release, is impaired at 7 and 14 days following CCI [18,32,20] and at 7 days following fluid percussion injury [19]. The current observation of reduced SV2A and SV2B abundance in the hippocampus at these time points may directly contribute to the impairments in SNARE complex formation after TBI. While the scope of the current study did not investigate the mechanisms contributing to reduced SV2 isoform abundances, this is an important question that needs to be addressed to first, understand how SV2 is reduced after TBI and second, to aid in identify an intervention to restore SV2 isoform abundance to uninjured levels. Previous work by Sheehan and colleagues demonstrated in cultured dissociated rat neurons that SV2A and VAMP2, an additional SNARE protein, are degraded in a neuronal activity dependent mechanism through the endosomal sorting complex required for transport (ESCRT) pathway with involvement of Rab35, while the synaptic proteins VGlut1 and synaptophysin remained unaffected [47, 67], representing a potential mechanism of presynaptic protein homeostasis. The SNARE protein SNAP-25 has been shown to be reduced by proteasomal degradation [68,69], and in the context of TBI, is a cleavage substrate of the injury-induced increased activity of calpain [70]. We have previously shown that multiple synaptic proteins, including VAMP2, alpha synuclein and SNAP-25, are reduced in experimental models of TBI in the days to weeks post-injury [18,19,20,32,46]. It is plausible that in addition to neuronal activity degradation, SV2 isoforms could be reduced by the pathological responses of TBI, including impaired axonal transport and altered mitochondrial function [71]. Considering the severity of injury with CCI, it is possible that cell loss and synaptic degeneration contribute to this reduction, but as we have previously shown, this is not the only contributing mechanism as reductions in alpha synuclein, VAMP2 and other exocytosis-mediating proteins do not fully align with the magnitude or time course of changes in the presynaptic protein synaptophysin [20,32,46], frequently utilized as an indirect marker of synaptic density. It is also plausible that reduced protein expression may reduce abundance as changes in SV2 isoform mRNA levels have been reported in multiple models of experimental TBI [37-39]. Additional work is needed to understand the mechanism by which SV2 isoforms are reduced, and previous studies suggest that altered ESCRT activity could contribute to reduced SV2 protein abundance. Collectively, the finding of reduced SV2 isoforms in the weeks after CCI may be the result of multiple underlying mechanisms, and additional work is warranted to better understand changes that can directly contribute to the presynaptic derangements of vesicular pool properties and impaired SNARE complex formation after TBI.

Reductions in the SV2A and SV2B abundances occur at time points of impaired acute motor function in the 5 days post-CCI and reduced performance in the hippocampal-dependent MWM task on days 9-14 post-CCI. Our observation of motor and cognitive impairments after CCI does not reveal novel neurobehavioral dysfunction as acute motor and MWM impairments in this two-week post-injury period are well-established; however, this observation supports that changes in SV2 isoforms may contribute to functional changes at time points in which these neurobehavioral impairments manifest. In the current study, we employed a traditional assessment in the hidden probe trial of time spent in the target quadrant for the 60 second testing period, as well as an enhanced target zone paradigm to further subdivide the target quadrant into the platform, inner ring, outer ring, and the remainder of the target quadrant. Analysis using the traditional assessment showed CCI-injured mice exhibited a significant decrease of time spent in the target quadrant. Assessment of probe performance using this subdivided zonal pattern revealed CCI-injured rats spent significantly less percentage of time in the outer ring, inner ring and platform during the total 60 second testing period. Similar analysis strategies using subdivided regions of the target quadrant have been utilized to help provide greater resolution of swim patterns and search strategies utilized by the subjects during the task [48-51].

Additional work is needed in the context of TBI, but the observations of increased developmental seizure susceptibility in SV2A and SV2A/B knockout animals [27,36], suggests that changes in SV2 abundance could contribute to underlying neuronal changes during acute or latency phases in the development of post-traumatic epilepsy. CCI has been shown to increase spontaneous seizures and increased epileptiform spiking via EEG monitoring in the months following injury [10,52-54,42], but the impact of CCI on SV2 has been unknown to date. Alterations in SV2A abundances have been a topic of interest in the context of temporal lobe epilepsy. In resected tissue from temporal lobe epilepsy patients, a 40 percent reduction in SV2A levels were observed in the neocortex by immunoblot [55]. Resected tissue from patients with hippocampal sclerosis showed reduced SV2A isoform expression in the dentate gyrus, CA3 and CA1, as compared to autopsy control tissues [56]. Comparatively in the same study using a rat model of status epilepticus (SE), reduced SV2A immunoreactivity was observed in the inner molecular layer and hilus at 1 day, with increasing loss in the dentate gyrus in the 1 week post-SE, culminating in progressive reductions in SV2A immunoreactivity throughout the hippocampus in the chronic phase at 6 to 8 month post-SE [56]. Our current findings show reduced SV2A in the molecular layer, hilus and granular layer, among other subregions at 2 weeks post-CCI, as well as reduced SV2B immunoreactivity in the molecular layer, similar hippocampal subregions exhibiting acute alterations in SV2A immunoreactivity in a model of SE [56]. However, despite these corroborating observations, additional work is needed to gain a better understand of SV2A changes with regard to temporal, regional, and in multiple models of TBI, as the magnitude and time course of SV2A changes has been shown to be influenced by the experimental model of epilepsy and species [57-59].

Importantly, SV2A was identified as the binding site for the anti-epileptic drug Levetiracetam [60,61]. Treatment with levetiracetam after TBI in the Operation Brain Trauma Therapy consortium has been one of the highest performing therapeutic interventions [62], and in multiple experimental TBI models levetiracetam has been shown to ameliorate synaptic and histological changes, kainic acid-induced seizure activity, and neurobehavioral dysfunction [63-65,62,66], further highlighting the need for a greater understanding of the changes in SV2 isoforms following TBI.

In summary, we demonstrate for the first time that TBI results in significant reductions in SV2A and SV2B abundance in the hippocampus and cortex in the weeks post-injury, at time points associated with impaired motor and cognitive function. In the cortex, reductions in SV2A were observed at 3 and 7 days post-injury, and SV2B reduced at 7 and 14 days post-injury. In the hippocampus, loss of SV2A and SV2B were observed by immunoblot at all time points assessed between 1 and 14 days post-injury. Hippocampal subregional analysis of SV2A immunoreactivity at 14 days post-injury revealed significant reduction throughout the hippocampus, while SV2B immunoreactivity reductions were observed primarily in the molecular layer. Provided the important role in regulation of the synaptic vesicular pool and formation of the SNARE complex, changes in SV2 isoforms may directly contribute to previously described alterations in intrasynaptic vesicular properties, impaired SNARE complex formation, and synaptic dysfunction after CCI, thus implicating changes in SV2 isoforms as contributors to dysfunction after TBI.

## Data Availability

All data generated during and/or analyzed during the current study are available from the corresponding author on reasonable request.
